# Brain organoids for hypoxic-ischemic studies: from bench to bedside

**DOI:** 10.1007/s00018-023-04951-0

**Published:** 2023-10-07

**Authors:** Romane Gaston-Breton, Auriane Maïza Letrou, Rifat Hamoudi, Barbara S. Stonestreet, Aloïse Mabondzo

**Affiliations:** 1 Université Paris Saclay, CEA, INRAE, Médicaments et Technologies pour la Santé (DMTS), Laboratoire d’Etude de l’Unité Neurovasculaire & Innovation Thérapeutique, 91191 Gif-sur-Yvette Cedex, France; 2 Research Institute for Medical and Health Sciences, University of Sharjah, P. O. 27272, Sharjah, United Arab Emirates; 3 College of Medicine, University of Sharjah, P. O. 27272, Sharjah, United Arab Emirates; 4 Division of Surgery and Interventional Science, University College London, London, UK; 5 ASPIRE Precision Medicine Research Institute Abu Dhabi, University of Sharjah, Sharjah, United Arab Emirates; 6 Departments of Molecular Biology, Cell Biology and Biochemistry and Department of Pediatrics, Women & Infants Hospital of Rhode Island, The Alpert Medical School of Brown University, 101 Dudley Street, Providence, RI 02905 USA

**Keywords:** 3D technology, Hypoxic-ischemic encephalopathy, Therapeutic trials, Translational research

## Abstract

Our current knowledge regarding the development of the human brain mostly derives from experimental studies on non-human primates, sheep, and rodents. However, these studies may not completely simulate all the features of human brain development as a result of species differences and variations in pre- and postnatal brain maturation. Therefore, it is important to supplement the in vivo animal models to increase the possibility that preclinical studies have appropriate relevance for potential future human trials. Three-dimensional brain organoid culture technology could complement in vivo animal studies to enhance the translatability of the preclinical animal studies and the understanding of brain-related disorders. In this review, we focus on the development of a model of hypoxic-ischemic (HI) brain injury using human brain organoids to complement the translation from animal experiments to human pathophysiology. We also discuss how the development of these tools provides potential opportunities to study fundamental aspects of the pathophysiology of HI-related brain injury including differences in the responses between males and females.

## Hypoxic-ischemic encephalopathy: a major cause of death and disability in the newborn period

Hypoxic-ischemic (HI) injury is the most common cause of brain damage in the newborn resulting in neurological abnormalities including cerebral palsy, intellectual deficits, cognitive developmental delay, and behavioral disorders [1]. The onset of these abnormalities can begin during pregnancy, labor, and delivery, or after birth. The etiology of brain injury has been widely reported. HI encephalopathy (HIE) can begin during pregnancy as a result of compromised placental perfusion, preeclampsia, maternal diabetes, congenital fetal infections, drug abuse, severe fetal anemia, or pulmonary disorders, along with a variety of other disorders [2]. HI-related brain injury originating during labor and delivery can result from umbilical cord occlusion, placental abruption, uterine rupture, excessive bleeding from the placenta, abnormal fetal position, prolonged late stages of labor, or maternal hypotension [3]. Finally, injury to the brain can also result from prematurity, severe lung or heart disease, infection, respiratory failure, or cardiac arrest after birth [4, 5]. HI-related brain injury has dramatic effects on the developing brain of the newborn as it affects the cerebrovasculature. The germinal matrix, a highly vascularized region of the developing brain located beneath the lateral ventricles, is vulnerable following adverse conditions including hypoxia, systemic hypo- and hypertension, and fluctuations in brain blood flow [6–8]. In addition, thin-walled cerebral blood vessels could be another source of vulnerability with structural immaturity or ongoing angiogenesis [9]. These observations suggest that cerebrovasculature responses after HI-related injury could contribute to injury in the neonate brain [8].

The incidence and outcomes of HIE depend upon the available resources. Low-income countries have a higher incidence of HIE compared with technologically advanced countries most likely because of limited resources and limited technology and personnel to provide neonatal intensive care [10]. In addition, less than adequate sanitation can contribute to a high incidence of neonatal sepsis [11]. The incidence of HIE is 1.5 per 1000 live births in developed countries and ranges from 2.3 to 26.5 per 1000 live births in developing countries [12]. HIE accounts for 23% of infant mortality worldwide and affects 0.7–1.2 million infants annually [13]. Twenty-five percent of infants who develop HIE at birth develop long-term neurological disabilities and 15% die [14]. There are approximately 125 million newborns with birth-related abnormalities, including 10 million who do not breath at birth, resulting in 1.2 million neonates potentially exposed to HI brain injury and approximately 0.5 million newborns, who are destined to develop neurodevelopmental abnormalities [1]. Although controversies remain regarding a definitive definition of HIE and classification of its severity, exposure of infants to HIE places a huge burden on families and society. Children with severe HIE may develop motor disorders, speech language problems, and vision and hearing impairment, which require long-term supportive care and supportive services. A report published in 2003 by Research Triangle Park (RTI International) and the Centers for Disease Control (CDC) estimated that lifetime costs in dollars totaled $2.1 billion for persons born in 2000 with hearing loss and $2.5 billion for persons with vision impairment [15]. These data emphasize the burden of these long-term consequences of HIE for the newborn and his or her family.

Sarnat and Sarnat [16] published a staging system in 1976 to identify specific parameters to validate the severity of what they termed “encephalopathy”. Nonetheless, it is often difficult to determine when or if HI results in neonatal encephalopathy. However, the term hypoxic-ischemic encephalopathy (HIE) is widely used, even though the diagnosis may be difficult to establish [17]. There is a need to improve the diagnostic criteria to facilitate parental counseling, diagnoses, prognostication, and treatment [18]. Classification of newborn encephalopathy is important because of the potential development of novel pharmacological neuroprotective agents that could serve as adjunctive or alternative agents to therapeutic hypothermia (TH), which is the current standard of care worldwide [19].

Long-term outcomes of HIE depend upon the severity, location of brain injury, and presence or absence of seizures. HIE is considered when the cord blood pH is less than 7.0 or 7.1, Apgar scores are less than or equal to 5 at 5 and/ or 10 minutes after birth and there is an apparent need for respiratory support [20]. The severity of HIE is estimated after the potential for HIE has been identified by serial neurological examinations using the Sarnat staging system. The severity is designated as normal, mild, moderate, or severe and is used to determine the necessity for treatment with therapeutic hypothermia. Mild HIE is diagnosed when the infant exhibits a hyper-alert state of consciousness, slightly decreased muscle tone, brisk deep tendon reflexes, irritability, and difficulty feeding, and sleeping, along with frequent episodes of crying. These symptoms can disappear within 24 h. Moderate HIE is present when the infant is lethargic, exhibits decreased spontaneous activity, hypotonia, a weak suck, and an incomplete Moro reflex. Finally, severe HIE is characterized by stupor or coma, flaccidity, decerebrate posturing, lack of spontaneous activity, absent suck, and absent Moro reflexes [21].

Although HIE remains an important cause of neurological disability and death in neonates, the only currently approved treatment is TH. This treatment requires that the infant be placed on a cooling mattress and that the temperature to be reduced to 33.5 °C within the first 6 h of life and maintained continuously at this temperature for 72 h of life. The infant is then slowly rewarmed after completion of the therapeutic cooling [22]. TH remains the only effective therapy for neonates with HIE and is now considered the standard of care worldwide. TH reduces cerebral metabolism by approximately 5% for each 1 °C of reduction in body temperature and is thought to reduce excitotoxicity, neuronal cell death, and improve neurological outcomes [23]. Other beneficial effects of hypothermia have been demonstrated including mitigation of blood–brain-barrier (BBB) dysfunction [24] associated with inhibition of neuroinflammation and reduction in specific markers of brain injury [25].

Whole-body hypothermia was investigated in a multicenter study [19] using an esophageal temperature of 33.5 °C for 72 h in full-term infants compared to a usual care control group. Infants with moderate or severe HIE exposed to whole-body hypothermia for 72 h exhibited reduced risks of death and disability, demonstrating the efficacy of TH in full-term neonates. The neurodevelopmental outcomes of the infants enrolled in the original trial were evaluated at 18–22 months of age [26]. The neurodevelopmental outcomes suggested that TH was safe and reduced the frequency and severity of brain injury after exposure moderate HIE compared with the normothermic control group, but severe HIE is not really improved by TH.

The ‘female advantage’, which suggests more favorable survival and neurodevelopment outcomes in female compared with male infants, has been previously reported for premature infants [27]. Smith et al. [28]*,* also showed that the female advantage extended to more favorable IQ performance in preterm infants at 5–12 years of age [28]. Hypoxia ischemia-related brain injury contributes to neonatal morbidities and is responsible for elevated mortality rates in both preterm and full-term neonates [13]. However, the types of brain injury differ in preterm and full-term infants [29]. HIE in full-term infants is associated with high mortality and morbidity rates [30, 31]. There is limited information comparing neurodevelopmental outcomes in male versus female infants [28], because most reports combine male and female outcomes and/or control for the male disadvantage in the analysis of developmental outcomes [32]. Therefore, this approach along with the limited information available on long-term developmental outcomes in male versus female full-term infants after exposure to HIE restricts the ability to draw conclusions regarding differences in developmental outcomes in full-term infants after HIE [28]. Available information has not yet identified the ‘female advantage’ in infants exposed to HIE with or without exposure to therapeutic hypothermia. However, these data could result from failure to detect differences in outcomes between male and female infants due to inadequate long-term follow up of a sufficient number of infants after exposure to HIE. Nonetheless, experimental findings in rodents support the contention that HI-related insults exhibit a similar male disadvantage with regards to brain injury potentially related to increased sex-specific inflammatory responses including greater infiltration of peripheral lymphocytes in males and elevated cytokine levels including tumor necrosis factor alpha [33]. Therefore, differences in inflammatory responses between males and females could potentially underlie the ‘female advantage’ in neonates [27].

## Potential molecular mechanisms of HIE

**Fig. 1:**
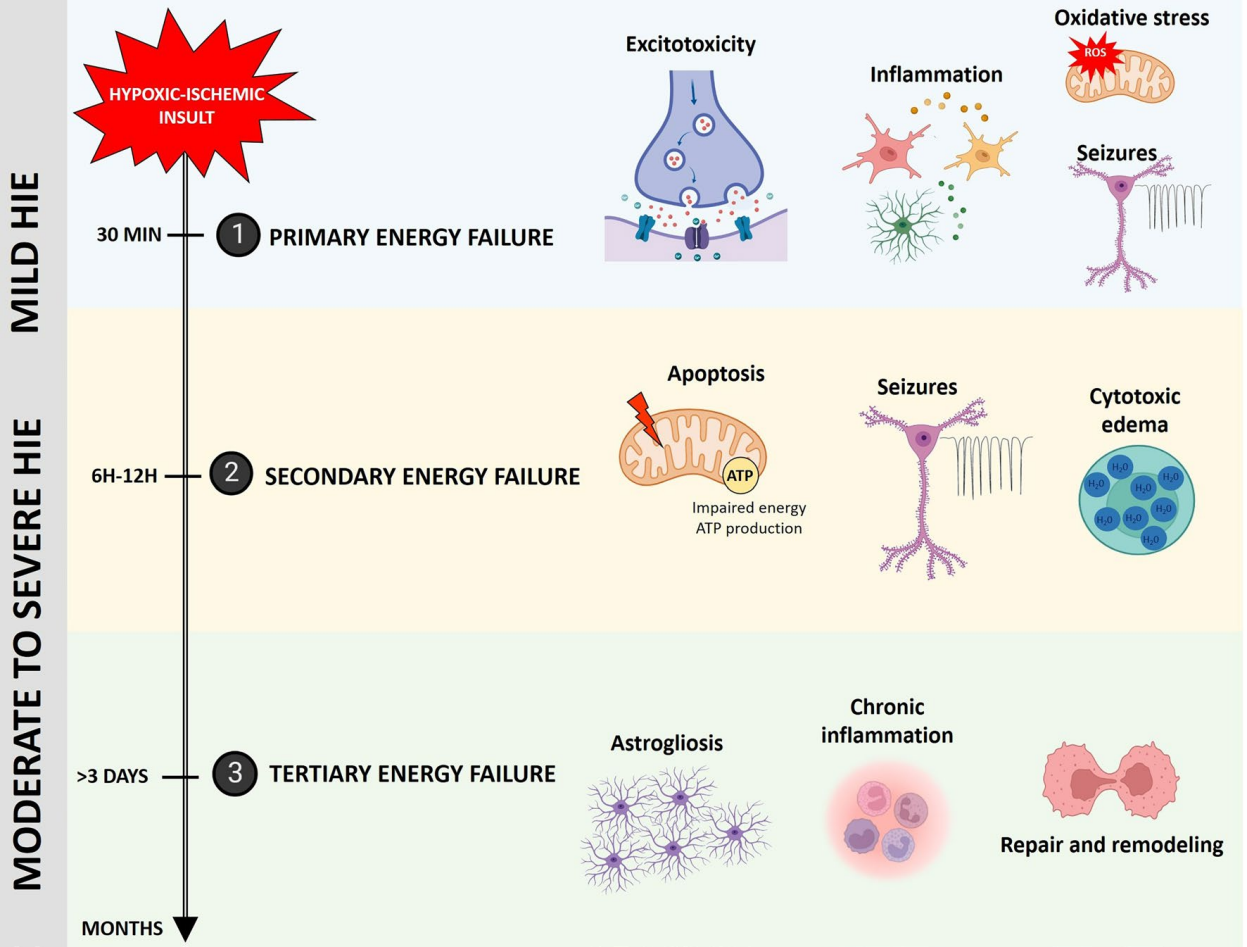
Schematic illustration of the pathophysiology of HIE. Hypoxic-ischemic (HI) insults result in anaerobic metabolism, because oxygen transport and tissue oxygenation are compromised in the fetal/neonatal brain resulting in depletion of adenosine triphosphate (ATP) and failure of the ATP-dependent Na + /K + pump. This event induces primary energy failure within minutes to hours with excitotoxicity, inflammation, oxidative stress, and reperfusion. Secondary energy failure occurs within hours to days with secondary mitochondrial failure including apoptosis and production of reactive oxygen species (ROS), seizures, cytotoxic edema, and hyperperfusion. The last stage is tertiary energy failure lasting days to months associated with astrogliosis, chronic inflammation, repair, and remodeling. These molecular cascades result in long-term neurological disabilities, such as developmental delay, sensory/cognitive abnormalities, learning disorders, seizures, and cerebral palsy. Adapted from [35] with BioRender.com

HIE is not a single event but a continuum of biochemical cascades, which lead to oxidative stress, inflammation, and neuronal cell death, over hours to days after the initial onset of injury (Fig. 1). The etiological event is oxygen and glucose deprivation in the developing brain, which results in anaerobic metabolism with ATP depletion and failure of ATP-dependent Na+/K+ pumps [34]. The initial injury results in cerebral metabolic alterations that can be divided into three phases. The initial stage is the acute phase known as “primary energy failure,” which occurs during the first minutes after the initial insult. This phase is characterized by anaerobic metabolism, oxidative stress, excitotoxicity, and neuronal cell death [35]. Excitotoxicity results from excessive exposure to the neurotransmitter, glutamate, or overstimulation of the AMPA, KA, and NMDA membrane receptors resulting in neuronal cellular injury or death. The second phase is the latent phase, which lasts from 1 to 6 h. A key factor in the latent phase is the development of neuroinflammation along with additional cascades including oxidative stress, excitotoxicity, and apoptotic and cell death pathways. The secondary phase occurs within 6–15 h and includes continued excitotoxicity, cytotoxic edema resulting from sodium and chloride ion accumulation along with fluid entering neurons and astrocytes, cerebral hyperperfusion, and secondary mitochondrial failure. Seizures often develop during this phase. Finally, the tertiary phase develops from weeks to months after the initiating event. The injured brain undergoes remodeling, astrogliosis, chronic inflammatory changes, and cell death during this phase [36].

Specific transcription factors produced during HI injury activate genes involved in anti-apoptosis, erythropoiesis, apoptosis, angiogenesis, and necrosis [37]. A key factor in this molecular cascade is hypoxia-inducible factor alpha (HIF1α), which mediates gene transcription to counterbalance the effects of the insults during HI injury. Mitogen-activated protein kinase (MAPK) is activated and phosphorylates HIF1α and HIF1β to stabilize the protein and dimerize the two subunits to form HIF1 during hypoxia [38]. The dimer acts on the hypoxia response elements to promote a variety of hypoxia responsive genes [39]. Therefore, the potential therapeutic window for HIE occurs between the latent and tertiary phases. However, as outlined above, therapeutic hypothermia is the only currently available efficacious treatment to attenuate the effects of HIE in full-term infants. This treatment is only partially effective as nearly half of infants with moderate-to-severe HIE newborn still survive with significant neurological disabilities or die despite treatment [40, 41]. Other therapeutic strategies such as treatment with erythropoietin have not proven efficacious [42]. Recent studies showed the role HIF1α and its downstream vascular endothelial growth factor (VEGF) in the damage of BBB integrity after ischemia and reperfusion [43, 44]. These studies showed that HIF1α inhibition reduces postischemic BBB damage in adult and neonatal rats and could imply that this neuroprotection is partially the result of blocking HIF1α signaling pathway and down-regulating VEGF activity. Targeting hypoxia-inducible factor and VEGF signaling could become a useful therapeutic approach for ischemic stroke.

**Fig. 2:**
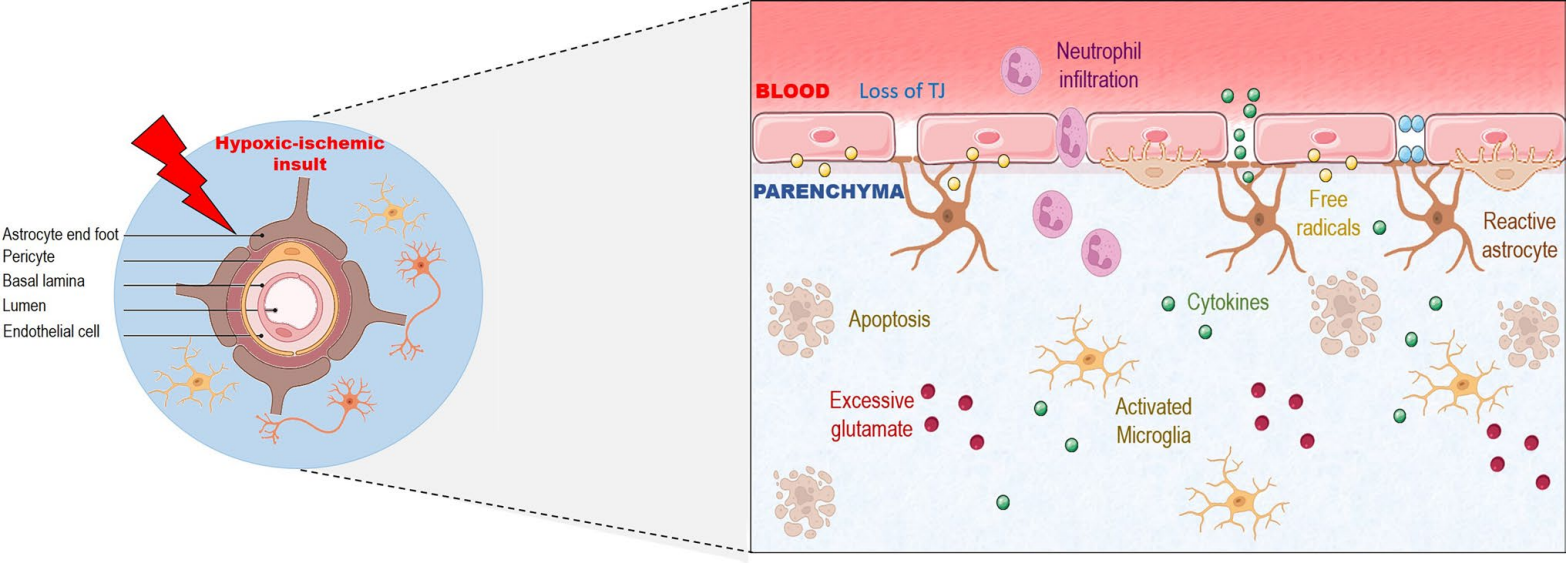
Blood–brain-barrier (BBB) disruption after a hypoxic-ischemic insults. BBB integrity is comprised after HI events associated with loss of tight junctions (TJ) between endothelial cells, neutrophilic infiltration, release of inflammatory mediators, such as cytokines, reactive astrocytes, excessive glutamate secretion, activated microglia, apoptosis, and free radicals. These molecular events result in increases in BBB permeability and disruption of the barrier. Created from BioRender.com

BBB dysfunction also potentially contributes to brain injury after exposure to HI injury, but has only been demonstrated in vivo and in vitro (Fig. 2). The BBB is an important physical/physiological barrier formed by the neurovascular unit (NVU), which is a multicellular structure, including astrocytes, pericytes, microglia, endothelial cells, extracellular matrix, and basement membrane. Tight junctions between adjacent endothelial cells are important structures, which selectively limit the passage of molecules into the brain parenchyma. Consequently, metabolites needed for neuronal activity enter via transporters and enzymatic activity [45]. Accordingly, the BBB maintains the homeostatic environment of the central nervous system (CNS). This barrier most likely plays a prominent role in pathophysiology of hypoxic-ischemic injury in neonates [46]. Exposure to HI injury potentially has many deleterious effects on the NVU and BBB function in the human adult brain. Previous work has shown disruption of the BBB after exposure to HI brain injury using a variety of methods and animal models [47, 48]. Exposure to HI affects all elements of the NVU. However, the endothelium is more susceptible to injury than the other cellular elements of the NVU including astrocytes and pericytes [49]. Compromise of the BBB after exposure to HI events could have important consequences including the development of vasogenic edema [50]. In addition, injury to the BBB could increase exposure of the brain parenchyma to inflammatory mediators, which activate matrix metalloproteinases (MMPs), which further accentuate inflammatory processes [51]. MMPs are also implicated in the proteolysis of the extracellular matrix protein and cleavage of tight junctions, which can result in edema and hemorrhage [52].

## Animal models to study HIE 

Many animal models have been used in an attempt to replicate the some of the components of HIE in newborn infants, although there is no model that completely replicates HIE in infants. Rodent, sheep, pig, and non-human primate animal models have been used to examine various aspects of the human disease [53]. The most important studies in animals are experiments in the ovine fetus, in which bilateral carotid artery occlusion is followed by various durations of reperfusion [54]. Studies of brain ischemia/reperfusion in the ovine fetus have demonstrated the neuroprotective efficacy of TH resulting in the worldwide use of therapeutic hypothermia in newborns exposed to HIE.

Several other animal models have been utilized to partially simulate HIE in human neonates and can roughly be divided into four categories [1]. The most widely used model is the Rice–Vannucci model [55]. This model facilitates the study of a relatedly large number of animals in comparison to the larger animal models [56]. Neonatal rodents at P7–P10 are exposed to unilateral carotid artery ligation with a recovery for approximately one and half hours, followed by exposure to 8% oxygen for 1–3 h at 36 or 37 °C. This model was originally described in rats but has been modified for use in mice with similar anatomical and behavioral effects [57]. The Rice–Vannucci model simulates a variety of anatomical lesions that are observed in human neonates with HIE including development of neuronal necrosis with injury to the cerebral cortex, hippocampus, thalamus, and basal ganglia [55, 58]. Other important lesions observed both in HIE in human neonates and in the Rice–Vannucci model include decreased cerebral blood flow [57], brain acidosis [59], decreased cerebral glucose uptake [60], white matter lesions [61], and prolonged inflammatory processes [62]. The Rice–Vannucci model has also been used to explore the effects of hypothermia and other potentially neuroprotective agents after exposure to HI injury in neonatal subjects. For example, Exendin-4, also known as exenatide, is a drug approved the U.S. Food and Drug Administration with the treatment of type 2 diabetes mellitus. It has been tested in combination of hypothermia in the context of HI brain injury in mice [63]. This preclinical study of combination of exendin-4 and hypothermia demonstrated an enhanced protection both in macroscopic injury score and regional infarct volume in a mouse model of HI. Another study investigated the effect of hypothermia on infection-sensitized neonatal HI brain injury on a rat model. In this lipopolysaccharide-sensitized unilateral stroke-like HI brain injury model, hypothermia was not neuroprotective in the hippocampus. The results of these studies in rodents suggest neuroprotection, as evidenced by smaller lesion volumes and improved performance in neurobehavioral outcome measures [64, 65].

The second category of animal model to simulate HIE is hypoxia without exposure to ischemia using an oxygen deprivation chamber. These models have not been studied as frequently as the Rice–Vannucci model but have been used to investigate hypoxia-related brain biochemical abnormalities [66]. The third category constitutes inflammatory models of perinatal brain injury in which intra-uterine infection often predisposes to preterm birth and brain injury [67]. Although the inflammatory models more closely simulate brain injury in premature infants, some characteristics of the inflammatory lesions are similar to those observed in HIE in the developing brain [68]. The fourth category is the non-human primate model in which monkey fetuses are exposed to umbilical cord occlusion [69]. Each of the animal model has advantages and disadvantages for the study of human disease. All of the models contribute to the investigation of mechanisms of HI injury and the potential development of therapeutic agents. Nonetheless, each model has limitations including species differences and timing of injury. The animal models cannot capture specific features of the human brain, including cognitive capacity, complex molecular processing, cellular responses, and unique genetic signatures. Unfortunately, many therapeutics which appear favorable from studies in rodent models have not proven efficacious, when subjected to human controlled trials.

## In vitro models to study HIE 

Several in vitro models have also been utilized to simulate HIE. Many studies have investigated hypoxia with 2D cellular culture models to examine neural progenitors derived from human embryonic stem cells [70] or rat organotypic hippocampal slices [71]. These 2D cell culture models can be divided into two categories for the study of HIE, oxygen deprivation, and oxygen-glucose deprivation (OGD). The first model exposes the cell culture to hypoxia alone. For example, human fetal neural stem cell-derived astrocytes have been exposed to oxygen deprivation [72]. In this study, neural stem cell-derived astrocytes were exposed to 2% oxygen as a model of moderate hypoxia and 0.2% oxygen as a model of severe hypoxia for 48 h to determine glutamate uptake after the two conditions of hypoxic injury. Changes in glutamate uptake were not observed suggesting that astrocytes play an important neuroprotective role during exposure to both moderate and severe hypoxic insults. Another study exposed mouse cortical neurons to hypoxia (95% N_2_, 5% CO_2_) for 24 h and examined the regulation of neuroglobin, a heme protein which enhances the supply of oxygen to neurons [73]. In vitro exposure of neurons to hypoxia increased neuronal neuroglobin expression. Furthermore, neuronal survival was reduced by inhibiting neuroglobin expression, suggesting that neuroglobin has an important role in neuronal survival after exposure to hypoxia.

OGD is a second category of in vitro studies widely used to simulate in vivo HI brain injury. For example, a study showed that polysaccharide 3 (LRP3) exerted neuroprotective effects on rat primary cortical neurons exposed to OGD [74]. Another study investigated the potential neuroprotective effects of stabilizing beta catenin after exposure of human neural progenitor cell cultures to OGD [75]. After exposure to 1% oxygen for 4 h, GSK-3beta inhibitors/beta-catenin stabilizers exhibited neuroprotective effects on human neural progenitor cells. Therefore, the in vivo animal models and in vitro cell culture systems described above have yielded and continue to provide a wealth of mechanistic information related to HI brain injury in adults and newborns. Nonetheless, many of these models do not contain some critical features specific to HI injury in the human brain. Consequently, additional models are needed to simulate human brain injury after exposure to HI.

2D monolayer cell culture is a useful tool to study behavior of specific cell type in brain disease; however, it is still relatively limited in providing a comprehensive understanding of complicated processes, such as cellular differentiation, tissue regeneration, and disease development [76]. In term of technical and experimental considerations, 2D models have lower costs, are moderately challenging, have lower time required, and have an artificial environment and a high reproducibility compared to brain organoid. In addition, vascularization is not possible for 2D models with monolayer; however, this aspect is important to consider in HI injury as endothelial cells are involved with an alteration of vascular integrity in pathophysiology of HIE [77]. However, vascularization and perfusion is highly possible in brain organoid using various methods, including 3D bio-printing strategy, organoids-on-a-chip, transplantation of organoid in vivo and perfusion after transplantation, fusion of vascular organoid, and brain organoid [78–80] Inflammation also plays a critical role in mediating brain injury induced by neonatal HIE [36]. Brain organoid can model a complex inflammatory system compared to 2D cell culture to study for instance human microglial phenotypes [81]

## Benefits of in vitro brain organoids for the study of human HIE

Four studies have already been completed with brain organoid culture systems relevant to HIE. These studies are described in Table 1 along with their advantages and disadvantages [82–85].

**Table 1:** Published studies describing the effect of hypoxic injury on brain organoid culture systems

References	Organoid type	Brain region modeled	Phenotypes of brain organoids	Hypoxic-ischemic modeling	Findings	Limitations
[82]	Human cortical organoid (dorsal forebrain specification)	Human forebrain	Cortical-like organization	1% or 8% O_2_ 5% CO_2_ for 25 days when brain organoids are 10 days old	This is the first study to demonstrate the effect of hypoxia on brain organoids and benefits of therapeutic agents such as minocycline	No microglia or vasculature in brain organoids
			Presence of neurons (immature neuron TBR1, neurons of cortical plate, synapses, glia, astrocytes and oRGC)		The authors showed that hypoxia has marked effects on forebrain cortex and ventral region	Hypoxia exposure on brain organoid at a very early neural development (when brain organoid are 10 days old)
					Minocycline prevents downregulation of neurogenesis by hypoxia	Minocycline effects have display different outcomes in clinical trial, discrepancies between preclinical and clinical data
					Minocycline may inhibit cell death induced by hypoxia	
[83]	Human cortical spheroid	Human cortex	Ventricular zone, subventricular zone and the cortical plate are delineated by the patterns of expression of PAX6, TBR2 and CTIP2	1% O_2_, 5% CO_2_ for 48 h at 37 °C ± 72 h of normoxia (21% O_2_, 5% CO_2_)	Platform to model hypoxic-ischemic encephalopathy of prematurity and as a model of a second trimester placental insufficiency	No existent immune cells, vascularized cells and interneurons inside the organoid
					The authors demonstrated that TBR2 + cells are particularly affected by oxygen deprivation	No inflammation component is captured here
						Oligodendrocyte, astrocyte and neuron network is not investigate here
[84]	Human cortical organoid	Human cortex	Presence of ventricle like structure aligned with a ventricular zone-like germinal regions and a and rudimentary cortical plate separated by a subventricular zone	3% O_2_, 5% CO_2_ for 24 h and then normoxia (21% O_2_, 5% CO_2_) until analysis	Implementation of HI model to investigate cellular behavior	Lack of vascularization
			Neuroepithelium with abundant radial glial cell (PAX6)		Identified high vulnerability of oRG progenitors	Lack of immune cells
			Cortical plate increases of thickness with immature neuron TUJ1 and neuron marker CTIP2		Observed a compensatory mechanism to replenish the stem cell niche after HI	Relatively early stage of cortical development
					Found a critical peri-hypoxia window during which differentiating neural populations are partially affected	
[85]	Human neural organoid	Human cortex	Expanded neuroepithelial morphology	1% O_2_ 5% CO_2_ for 48 h at day 84 for brain organoids and then 24 h at 21% O_2_ 5% CO_2_ for reoxygenation step	This method can be used to model neural development and neurodegenerative disease	Lack of vascularization
			PAX6 enriched apical progenitor zones surrounded by TUJ1 immature neuron		New platform for drug screening for clinical assay for brain ischemia	Immune cells absent and no inflammatory component captured here
			Presence of NeuN and MAP2 mature neuron		After HI, there was a decrease in specific cortical progenitors	No cortical circuits investigated

*oRGC* outer radial glia cells, *Pax6* paired box protein 6, *TBR2* T-box brain protein 2, *CTIP2* COUP TF1-interacting protein 2, *Tuj1* Class III beta tubulin, *MAP2* microtubule associated protein 2, *NeuN* neuronal nuclear antigen

Brain organoids represent the next generation of promising novel in vitro models for the study of hypoxia-related injury in the human brain. Lancaster et al. first initiated the development of brain organoids for the modeling of microcephaly in 2013 [86]. Their 3D model of the human brain was developed from human pluripotent stem cells, which simulate specific brain regions in vivo. These brain organoids reproduced the cytoarchitecture of the cerebral cortex and have the ability to develop multiple brain regions and cellular types. The 3D structures of the brain organoids facilitate the proliferation, migration, and differentiation of the different cell types. Astrocytes, neurons, oligodendrocytes, and microglia have been generated within brain organoids depending on the use of particular protocols [87]. Cellular identification appears at specific periods during the development of brain organoid cultures [88]. For example, markers of astrocytes and microglia have been shown to increase after 1 month, whereas oligodendrocytes gradually emerge after the brain organoids have been cultured for 3 months [88]. Consequently, cellular identities, neuronal activity, and cellular interactions are a function of the duration of time that the brain organoids remain in culture.

Brain organoid cultures have been shown to replicate early in vivo brain development remarkably well from the middle to the end of the 8–10 weeks of gestation (GW) [89]. Some reports have demonstrated that the in vitro development recapitulates in vivo development up until approximately 19–24 weeks of gestation, suggesting that this model is particularly relevant to human fetal brain development [90, 91]. Similarities have been shown to exist between in vitro neocortical cultures and early human neocortical development with respect to their temporal development and gene expression [92]. In addition, cortical organoids have recently been shown to mimic dynamic neural networks similar to those of premature neonates at approximately 28 week gestation [93]. Consequently, the recent development of brain organoid technology facilitates an approach to human-specific neurodevelopmental processes with respect to their transcriptional signatures, recapitulation of the dynamic cytoarchitectural development, and functional electrophysiological maturation [94].

The considerations outlined above suggest that 3D organoid culture models could provide novel insights into cellular mechanisms and responses, along with specific biomarkers related to signature of human HIE. Recent studies have examined brain organoid cultures to simulate neonatal encephalopathy using several different approaches. Several recent studies have examined the neuroprotective efficacy of minocycline to attenuate hypoxic insults using human brain organoids [82]. Minocycline is a second-generation semi-synthetic tetracycline derivative that has been shown to exhibit neuroprotective properties [95]. HIE was simulated by exposure of the brain organoid cultures to hypoxia (1% and 8% oxygen) using a hypoxia chamber from day 10 of the neural organoid culture induction up until to 25 days of exposure to hypoxia. Thereafter, the cultures were exposed to minocycline for 72 h. However, this protocol induced hypoxia at a very early stage of brain organoid development that could potentially limit the relevance of the hypoxic exposure to HIE in the human full-term human infants [89, 92]. Minocycline has been evaluated in hypoxic brain injury but also in neurodegenerative and psychiatric diseases [96, 97]. This molecule displays effective antioxidant and anti-inflammatory effects in preclinical studies in animal models but not in clinical studies [98]. Matrigel was not used during the culture of organoids in this study as well as bioreactors or extrinsic signaling molecules, which limit neuroectoderm expansion and appropriate differentiation of the cellular elements into the neuronal population. The gap between preclinical and clinical data suggests that these systems need to be carefully evaluated.

Hypoxic injury was also induced in 3D human neural organoid on day 84 of culture by exposure to low oxygen conditions (1% of oxygen) for 48 h [85]. After exposure to hypoxia, the neural organoids were placed in a chamber containing 21% O_2_ and 5% CO_2_ for 24 h to simulate reoxygenation. The organoids in this study represented organized human neural organoids recapitulating human cortical plate development, thereby increasing the relevance of the model to human neonates exposed to hypoxic conditions. Hypoxic brain injury was characterized by disruption of neuronal cell components after exposure to hypoxia. Moreover, reoxygenation resulted in neuronal cell proliferation but could not reconstitute appropriate neuronal maturation. In addition, an attempt was made to expose the neural organoid cultures to OGD. However, OGD resulted in severe damage, which could not be reversed upon exposure to reoxygenation.

Hypoxic injury was also examined at 28 days of brain organoid culture by applying 3% O_2_ and 5% CO_2_ for 24 h followed by normoxic conditions (21% of O_2_ and 5% CO_2_). These study conditions resulted in injury to the outer radial glia (oRG) progenitors and other neuronal cells, and compensatory mechanisms to reconstitute stem cells after HI [84]. Finally, hypoxic brain injury was used to investigate the effects of hypoxia using three-dimensional human brain region-specific organoids. Oxygen deprivation (1% O_2_ and 5% CO_2_) for 48 h altered corticogenesis because of damage to intermediate progenitors as a result of responses in unfolded proteins [83].

Overall, these studies tested different conditions of hypoxic-ischemic injury by changing the percentage of oxygen, exposure time, and age of brain organoid exposed and recovery condition. To study HI pathophysiology through this 3D technology, there are number of criteria to consider. The first one is the age of brain organoid exposed to injury. HIE occurs during prenatal, intrapartum and postnatal period in neonates, so to compare with in vivo situation, human brain organoid must display similarities, including cellular distribution and organization, physiological structure, electrical activities, and neuronal networks. In fact, there is a changing organoid physiology over time in culture and age of brain organoid must be chosen carefully to allow cell maturity, full functionality and to enable for the target cell types to differentiate [88]. Neurons start to appear in earlier time of culturing (around 1 month). Marker of astrocytes and microglia increases from 1 month, while oligodendrocytes gradually emerge from 3 months of culturing. At 5 and 6 months old, there are more complex neuronal networks with the presence of periodic oscillatory activity and synaptic plasticity. To study HIE, brain organoid need a minimum of 1 month of maturation in culture to display neuronal network, cell type diversity and to allow researcher to observe cell’s behavior. The second criteria to consider is percentage of oxygen for hypoxic injury on brain organoid. When hypoxic injury happen during prenatal period, the percentage of oxygen deprivation is not known. Different percentage of oxygen need to be tested on brain organoid and validate using cell marker (neurons, astrocytes, and microglia) or more specific marker such as HIF1α a major transcriptional regulator of the cellular response to low oxygen. The third criteria is additional implementation of glucose deprivation to model ischemia-like conditions in vitro [99]. One study tried OGD on brain organoids [85] and led to severe damage including a decrease in size, a loss of layer structure, and gene expression TUJ1 and PAX6. The last criteria is to add a reoxygenation step after hypoxic-ischemic injury, normoxia with 21% of oxygen. This step can model reperfusion/reoxygenation of neonates after hypoxic-ischemic injury. This simulation of reoxygenation can be really interesting to study behavior of neurons and the other cell types after reoxygenation.

These early studies outlined above suggest the relevance and feasibility of using brain organoid cultures as a model to simulate some of the characteristics related to HIE in the human neonate. Organoid cultures could facilitate understanding of the effects of hypoxic insults on specific cellular responses, molecular mechanisms, and the behavior of brain signal transduction pathways resulting in injury and/or recovery from injury.

## Challenges, limitations, and solutions for brain organoid cultures

Given the considerations summarized above, it appears that the 3D cerebral cortical organoid culture system has great potential to facilitate the translational study of brain diseases such as HIE in the newborn and to provide detailed preclinical mechanistic data. Nonetheless, this methodology also entails considerable challenges for its implementation and verification (see Table 2). The first limitation is the absence of a microvasculature network containing native blood cellular elements within the cultured brain organoids. Although there is sufficient oxygen and nutrients near the surface of the organoid cultures, their availability becomes limited in the deeper regions in the organoid cultures. Consequently, a gradient potentially exists within the organoid cultures resulting in acidification and release of inflammatory contents predisposing to apoptosis potentially, which damage neighboring cells via adherent junctions at the deeper culture levels and leads to necrotic core development. Limits in diffusion could result in reductions of proliferation and structural disorganization when cultures are maintained for longer durations [100].

**Table 2:** Challenges in brain organoid model

Limitations	Consequences	Approaches
Absence of microvasculature network	Oxygen and nutrient availability become limited in the deeper region. This limitation will lead to formation of a gradient within brain organoid with acidification, inflammation, and apoptosis	Co-culture with endothelial cells, invasion into the brain organoids [103] Transplantation of brain organoids inside the cerebral cortex of immunodeficient mice[105] Use of endogenous endothelial cells that overexpress the transcription factor human ETS variant 2 (ETV2) [106] Generation of blood vessels within organoids using fusion of vascularized organoids with brain organoids [107] Development of neurovascular organoids using a 3D-printed microfluidic chips [126]
Absence of microglia cells	Lack of specific cell types and their microenvironment	Human iPSC-derived microglial have been cultured along with brain organoids with migration into the interior of the brain organoids [108] Induction of oligodendrocytes progenitors and myelinating oligodendrocytes in cortical spheroids by exposure to the oligodendrocytes growth factors PDGF, IGF-1 and T3 [127] Differentiation of hiPSC cells into 3D neural spheroids to model the development of human oligodendrocyte lineage cells alongside neurons and astrocytes [128] Generation of microglia-containing brain organoids by co-culturing hPSCs-derived primitive neural progenitors cells and primitive macrophage progenitors under 3D conditions [129]
Poor spatial organization, complexity, maturation, cortical folding, and gyrification inside brain organoids	Different cell organization inside brain organoid compared to human brain, less complexity of cell type, less maturation and no cortical folding	Generation of a “pseudo folding” by inducing neural progenitor overgrowth via knockout PTEN or by microchips [111, 112, 130] In situ generation of human brain organoid on a micropillar array with specific features of neuronal differentiation, brain regionalization, and cortical organization [131] Development of a lipid-bilayer-supported printing technique to 3D print human cortical cells in Matrigel [132] Engineering 3D brain organoid derived from hiPSCs using organ-on-a-chip technology revealing well-defined neural differentiation, regionalization, and cortical organization [133]
High variability within and between organoid batches	Reduce reproducibility of data	Development of a new matrix with human brain extracellular matrix which can promote structural and functional maturation of organoids [115]

Several different techniques have been attempted to vascularize organoid cultures to minimize the adverse effects of these gradients [101]. Blood vessels are derived from mesodermal tissue, whereas neurons are from ectodermal tissue [102]. Therefore, it is difficult to initiate cell cultures from two different germ layers simultaneously [102]. Several studies have attempted to vascularize 3D organoid cultures by co-culturing brain organoids with endothelial cells. This process results in vasculature cover or invasion into the brain organoid cultures [103, 104]. A limit of this strategy is that vascularization does not lead to a functional BBB in this model.

Another strategy is to transplant the brain organoids into the cerebral cortex of immunodeficient mice. This procedure results in invasion of the host blood vessels into the brain organoids with active blood flow perfusion [105]. However, this technique results in a half-mouse/ half-human model and brain organoid are not independently vascularized.

An alternative protocol utilized endogenous endothelial cells that overexpressed the transcription factor human ETS variant 2 *(ETV2),* which induced differentiation of pluripotent stem cells into endothelial cells during brain organoid culture initiation [106]. A recent study generated, blood vessels within organoids using transient mesodermal inductions, vascular progenitor, and endothelial cell inductions and at the end incubation with neurotrophic reagents [107]. After this procedure, vascularized organoids were infused into the brain organoids. Interestingly, the brain organoids became fused with the vascular tissue. The new structures had properties similar to those of the BBB and actually exhibited tight junctions. However, the generations of cellular and plasma components of blood also need to be established. Although there are several novel strategies that appear to be able to vascularize brain organoids in support of the complex cellular network in brain organoids, the development of a microvasculature network containing the cellular and plasma constituents of blood remains a considerable challenge.

Several other challenges include the necessity for astrocytes and oligodendrocytes to simulate defined cortical layers similar to the in vivo cerebral cortex. The microenvironment needs simultaneously to contain all cell types present within normal brain. Human iPSCs-derived microglial have been cultured along with brain organoids and shown to adhere to, and migrate into the interior of the brain organoid [108]. Other challenges include obstacles to spatial organization, complexity, maturation, appropriate cortical folding, and gyrification as observed in gyrencephalic mammals [109, 110]. The lack of appropriate cortical folding in the brain organoids could result from the fact that the cultures do not reach a developmental stage, at which gyrification is present [110]. There have been attempts to generate a ‘pseudo folding’ into brain organoids by inducing neural progenitor cells (NPC) overgrowth via knockout PTEN or by microchips [111, 112]. Moreover, there is considerable variability in the 3D model within and between organoid batches, thus limiting reproducibility, which impairs the validity of the data generated with this model system [113]. The use of Matrigel increases this variability, as most organoids are cultured with Matrigel, which come from Engelbreth–Holm–Swarm mouse sarcoma cells, which is rich in collagen, laminin, proteoglycan, and other extracellular matrix protein. These factors make it difficult to elucidate the type of structure and function of organoids and limit the use of organoids in clinical transplantation [114]. A new matrix has been developed, which contains brain extracellular matrix, and can promote structural and functional maturation of human brain organoids [115]. This extracellular matrix is complicated to obtain, because it is human brain tissue and this makes it difficult to use.

## Where promising discoveries meet their demise: brain in a dish as a novel tool to bridge the translational gap for HIE

“The translational science spectrum represents each stage of research along the path from the biological basis of health and disease to interventions that improve the health of individuals and the public” according to the U.S. National Center for Advancing Translational Sciences (NIH).

There are five important steps before clinical application can be achieved. The first is basic science research with preclinical and animal studies [116]. (1) The aim of this step is to define the pathobiological mechanisms and targets, and to produce relevant therapeutic agents and determine regulatory interactions. The second step is translation to human clinical trials. (2) The goals of phase one clinical trials are to determine safety, proof of mechanism(s) and of concepts, new methods of diagnosis, treatment, and prevention. The third step is the initial clinical trial. (3) Phase 2/3 clinical trials address the dose selection, proof of efficacy, and patient safety. The aims of this stage are to perform controlled trials resulting in efficacious therapeutics, to determine the benefit/risk profile, and to generate health economic data. There is a gap between stages one and three that has been dubbed the “Valley of death”. Therefore, it remains critical for basic and clinical scientists to achieve optimal communication during the initial development of clinical trials [117]. (4) Phase four represents translation into clinical practice through further clinical trials and outcomes research, in which therapeutics are delivered in a timely fashion to the appropriate clinical population, along with post-marketing safety evaluations, and the potential evaluation for supplementary indications. (5) The final stage is translation to the community with societal benefit. This includes translation of new data into clinical decision-making.

More relevant accurate models are required to convert novel findings obtained from basic science research into clinically relevant therapeutics. Consequently, there is a critical need for improved efficacy and evaluation, for enhanced predictive assessment, and translation of data to bring innovations more rapidly from bench to bedside [118]. Brain organoids could potentially help bridge the gap between novel scientific discoveries and clinical application [119]. This technology is a promising platform to study brain diseases, such as HIE and to investigate their underlying molecular mechanisms. As discussed previously, brain organoids have some limitations such as a necrotic core and hypoxia in deeper region due to a lack of circulating nutrients and oxygen. However, there are some solutions that deal with this limitation, which can bias data interpretation in a hypoxic context. In a study published in 2021, [120] investigated a new method to avoid apoptosis and hypoxia in the organoid core: they mechanically cut 70-day-old human cortical organoids with a scalpel blade into 2 or 4 pieces [120]. After 7 days of culturing these pieces of brain organoids, they evaluated HIF1a, which was not expressed in cut organoids with less pro-apoptotic marker such as Bcl-2 and Bax. Another possibility is to use cerebral organoid at the air–liquid interface, which lead to an improved survival and maturation [121]. Discovery of specific biomarkers though the brain organoids platform could facilitate drug development [122]. Brain organoids could potentially facilitate novel neuroprotective strategies tailored to enable personalized medicine. This methodology could accelerate the drug development pipeline. Personalized medicine could compensate for inter-individual differences and different patient phenotypes, and facilitate personalized diagnosis along with prognostication [123]. Personalized medicine could also elucidate sex differences and allow for specific patient traits. “Biological sex” is a key variable in biomedical research that needs to be considered in all experiments [124].

## Concluding remarks and future perspectives

HI injury is a global issue accounting for 23% of infant mortality worldwide [13]. Hypothermia is the only therapeutic strategy approved for full-term infants that are exposed to HIE. However, this therapy is only partially protective and there is an urgent need to develop adjunctive [100] neuroprotective agents to improve outcomes after exposure to HIE. Brain organoid cultures have the potential to provide translational data and to accelerate the transfer of therapies from basic science to clinical medicine. This 3D model could contribute to neurological research and represent an interesting method to study disease states, and to evaluate new therapeutic agents [125]. It will be an important challenge to improve this in vitro platform through: implementation of different cell types found in the human brain, the modeling of the brain microenvironment, establishment of effective vascularization, and spatial organization specific to brain regions in human subjects. This platform will also be a useful tool for the study of sexual differences reported in HIE, using iPSCs from human males and females in brain organoids.

## Data Availability

All data are available in PubMed website.
